# Inverse Laplace transform and multiexponential fitting analysis of T2 relaxometry data: a phantom study with aqueous and fat containing samples

**DOI:** 10.1186/s41747-020-00154-5

**Published:** 2020-05-07

**Authors:** Georgios S. Ioannidis, Katerina Nikiforaki, Georgios Kalaitzakis, Apostolos Karantanas, Kostas Marias, Thomas G. Maris

**Affiliations:** 1 Foundation for Research and Technology-Hellas (FORTH), Institute of Computer Science (ICS), Computational Bio-Medicine Laboratory (CBML), N.Plastira 100, Vassilika Vouton, Heraklion, GR-70013 Crete, Greece; 2 School of Medicine, University of Crete, Heraklion, Greece; 3 Department of Medical Physics, University of Crete, Heraklion, Greece; 4 Department of Medical Imaging, University Hospital, Heraklion, Greece; 5 Department of Electrical & Computer Engineering, Hellenic Mediterranean University, Heraklion, Greece

**Keywords:** Inverse Laplace transform, Magnetic resonance imaging, Multiexponential non-linear fitting, Multicompartment T2 relaxometry, Phantoms (imaging)

## Abstract

**Background:**

The inverse Laplace transform (ILT) is the most widely used method for T2 relaxometry data analysis. This study examines the qualitative agreement of ILT and a proposed multiexponential (Mexp method) regarding the number of T2 components. We performed a feasibility study for the voxelwise characterisation of heterogeneous tissue with T2 relaxometry.

**Methods:**

Eleven samples of aqueous, fatty and mixed composition were analysed using ILT and Mexp. The phantom was imaged using a 1.5-T system with a single slice T2 relaxometry 25-echo Carr-Purcell-Meiboom-Gill sequence in order to obtain the T2 decay curve with 25 equidistant echo times. The adjusted *R*^2^ goodness of fit criterion was used to determine the number of T2 components using the Mexp method on a voxel-based analysis. Comparison of mean and standard deviation of T2 values for both methods was performed by fitting a Gaussian function to the ILT resulting vector.

**Results:**

Phantom results showed pure monoexponential decay for acetone and water and pure biexponential behaviour for corn oil, egg yolk, and 35% fat milk cream, while mixtures of egg whites and yolks as well as milk creams with 12–20% fatty composition exhibit mixed monoexponential and biexponential behaviour at different fractions. The number of T2 components by the Mexp method was compared to the ILT-derived spectrum as ground truth.

**Conclusions:**

Mexp analysis with the adjusted *R*^2^ criterion can be used for the detection of the T2 distribution of aqueous, fatty and mixed samples with the added advantage of voxelwise mapping.

## Key points

- T2 relaxometry provides tissue/material specific information.
- The multiexponential method requires no assumption for the number of T2 components, can provide T2 distributions in accordance to inverse Laplace transform results, and has the advantage of providing voxelwise T2 mapping.
- Fat samples showed pure biexponential T2 decay.

## Background

One of the best established biomarkers for tissue characterisation is transverse magnetisation (T2) relaxometry, utilised for a large variety of clinical [1–3] and also non-clinical applications [4–6] as it provides information from the inner structure of the imaging object and is also independent of reader, pulse sequence or imaging device [7]. Current clinical applications of T2 relaxometry include assessment of myelin in the brain, collagen or oedema/inflammation in the articular cartilageand of fat and iron content in the liver and heart [8]. T2 relaxation curve is affected by the tissue free water content, fraction of water bound to molecules and macromolecules, local tissue temperature, tissue fat content, presence of paramagnetic particles and pH value [4]. Consequently, an appropriate analysis of T2 decay may reveal information stemming from the inner structure of tissue or material at a submillimetre scale.

Free water exhibits pure monoexponential decay with long T2 values while water in tissue bound to lipids and proteins has a different relaxation behaviour with shorter T2 values [9–11]. Materials mimicking adipose tissue relaxation such as corn oil present biexponential decay with a shorter and a longer T2 component, indicating the presence of two proton components in the fatty acid chains [12]. Clinical T2 relaxometry sequences measure signal from aqueous or fatty components indiscriminately within a single voxel. Thus, acquired T2 relaxometry curve is the overall result of the summation of the signal coming from both water and lipid protons in a certain voxel. In order to gain insight into voxel composition, it is important to decompose the voxel signal into its distinct T2 components and their calculated T2 value as descriptive features of its composition.

The focus of this study is the characterisation of fat and water containing phantom samples based on two different relaxometry methods, the inverse Laplace transform (ILT) [13] and a proposed multiexponential (Mexp) method. This in turn will serve as a preliminary step to evaluate the agreement of the proposed Mexp method with the well-established ILT method.

The Mexp method proposed herein requires no a priori assumption on the number of components (one or two) but rather uses the objective T2 criterion for the optimal choice regarding the number of T2 decaying exponential components*.* This in turn reduces bias related to the assumption of either one or two exponentials for the solution compared to conventional mono or bi exponential fitting techniques. The added advantage of the proposed method over ILT is the capability of voxelwise T2 mapping which is very important when considering the implementation of this method to the study of heterogeneous materials or tissue. It is worth noting that even for a single voxel, the ILT method returns a T2 distribution (vector outcome for each voxel) rather than a discrete T2 value. Similarly, for a region of interest (ROI)-based study, the ILT result is a distribution of T2 values over the whole ROI area, the metric of interest being the shape and the number of the distinct T2 peaks. Taking into account that mapping tissue heterogeneity is very important for oncologic and neurologic studies and voxelwise parametric mapping can improve the diagnostic value of T2 relaxometry. To the best of our knowledge, a composite phantom study combining ILT and Mexp T2 relaxation methods as well as a voxel-based multicompartment description of T2 relaxation properties has not yet been performed.

## Methods

### Imaging protocol

Magnetic resonance imaging was performed on a 1.5-T scanner (Vision/Sonata hybrid System, Siemens, Erlangen, Germany). The T2 relaxometry protocol consisted of a two-dimensional single-slice multi-echo spin-echo approach based on a two-dimensional multi-echo Carr-Purcell-Meiboom-Gill spin-echo sequence with alternating 180° radiofrequency pulses under the phase-alternating phase-shift scheme [14]. The final result was a single-slice, proton density-to-T2-weighted sequence with a repetition time of 2,500 ms, 32 equidistant spin echoes, echo spacing of 7.3 ms and last echo at 233.6 ms). A field of view of 230 × 144 mm with a rectangular reconstruction matrix (256 × 160 pixels) was chosen. A selective refocusing radiofrequency pulse scheme was utilised for eliminating stimulated echoes [15].

### Data preprocessing

In the multi-echo spin-echo/phase-alternating phase-shift sequence, the first echo signal is not accurate because of *B*_1_ field imperfections [16, 17] and is usually either extrapolated or ignored. In this study, we did not correct for the first echo as extrapolation process requires the choice of a proper model and this would in turn introduce bias to the next step of monoexponential or biexponential fitting model selection. Therefore, signal at the first echo time (at 7.3 ms) was excluded. This confounds the minimum bound for T2 estimation, which was chosen at 14.6 ms for the present study.

### Phantom study

**Fig 1:**
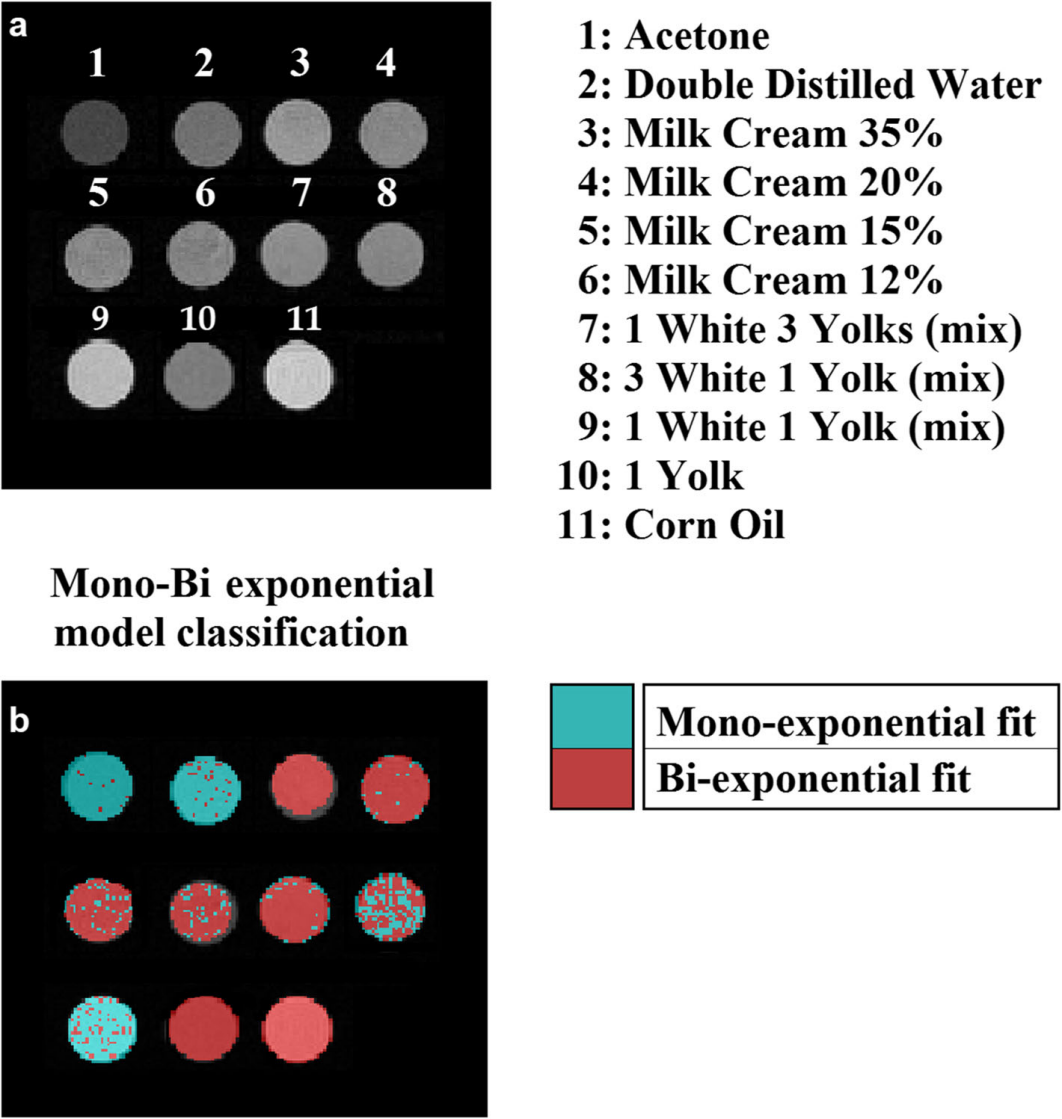
**a** T2-weighted image of eleven samples of aqueous, fatty, and mixed composition. **b** Multiexponential/biexponential classification of each sample based on the ${\overline{R}}^2$ criterion

The in-house built phantom [18] was composed of eleven vials of 2 cm in diameter, filled with samples of both aqueous, fatty, or mixed composition. The phantom was left overnight in the scanning room to obtain room temperature of 20 °C. All vials were placed in a phantom holder, and their positions as well as an axial T2-weighted image (echo time 26.8 ms) are presented in Fig. 1. For all samples, we expected T2 values no greater than 2,500 ms [19] which was set as the upper bound of the Mexp method. At a later stage of our study, we also prepared a sample of pure egg white for purposes of verification of our results regarding ILT performance at low fat content compositions.

### T2 relaxometry

All numerical calculations concerning Mexp T2 relaxometry were implemented in Python 3.5 (www.python.org) apart from the ILT method which was implemented in Matlab 2015a (Mathworks, Natick, MA, USA). The graphical user interface and result visualisation were accomplished by the use of PyQt4 and PyQtGraph (www.pyqtgraph.org) libraries respectively. The mathematical framework and technical details for the T2 relaxation are presented below.

### Inverse Laplace transform method

A continuous magnetic resonance relaxometry signal *y*(*t*) is of the form:

1 $$ y(t)=\underset{0}{\overset{\infty }{\int }}{e}^{-\frac{t}{T_2}}\ f\left({T}_2\right)d{T}_2+e(t)\kern0.5em $$

where *T*_2_ denotes the relaxation time, *f*(*T*_2_) represents the amplitude of the corresponding component, and *e*(*t*_j_) is the instrumentation noise. After the discretisation of (1), the goal is to extract the distribution of the *f*(*T*_2_) amplitudes through (2):

2 $$ g\left({t}_j\right)=y\left({t}_j\right)-e\left({t}_j\right)=\sum \limits_{i=1}^N{e}^{-\frac{t_j}{T_{2i}}}\ f\left({T}_2\right)\kern0.5em $$

in equation (2), the index *j* stands for the number of echoes. In matrix notation, the main aim is to solve the linear system *g* = *Af* where ${A}_{\mathrm{ji}}={e}^{-\frac{t_j}{T_{2i}}}$ is the discrete Laplace transform and *g*(*t*_*j*_) = *y*(*t*_*j*_) − *e*(*t*_*j*_). For the purpose of our analysis, *e*(*t*_*j*_) was considered to be the vector containing the mean background noise for every TE. The problem thus is to find the vector ***f*** by minimising (3) below:

3 $$ \boldsymbol{f}=\mathit{\arg}\underset{f\ge 0}{\min }{\left\Vert g-A\boldsymbol{f}\right\Vert}_2^2 $$

Taking into consideration that the inverse Laplace transform is a highly ill-posed problem and therefore intrinsically affected by numerical instability, its solution may not be unique [1]. To address this limitation, a penalty term (*a*) is introduced [20] to increase stability in the inversion as illustrated in (4). This technique is known as Tikhonov regularisation:

4 $$ \boldsymbol{f}=\mathit{\arg}\underset{\boldsymbol{f}\ge 0}{\min }{\left\Vert g-A\boldsymbol{f}\right\Vert}_2^2\mathrm{euq}+a\ {\left\Vert \boldsymbol{f}\right\Vert}_2^2 $$

Additionally, for the phantom study, signal *y*(*t*) was the mean ROI signal from all voxels assigned as a certain sample. Lastly, the Matlab “fminsearch” function equipped with the Nelder-Mead simplex direct search was used to obtain the vector ***f*** with*a* = 0.01. The selection of the proper *α* was based on Morozov’s discrepancy principle [21] stating that the value of *α* is chosen such that the norm of the residual ${\left\Vert g-A{\boldsymbol{f}}_{\mathbf{s}}\right\Vert}_2^2$ (***f***_**s**_: solution vector) equals the norm of the error term *e*(*t*). Thus, after executing the ILT on several instances, we concluded to *a* value of the order of magnitude of 10^−2^. The ILT method was used as a reference method to test the results of the proposed Mexp methodology in terms of number of distinct *T*_2_ components and measured *T*_2_ values.

### T2 multiexponential analysis

Supposing an MR signal *S*(*t*_*j*_) measured at echo times *t*_*j*_ (*j* = 1,  2, …, *K*), the decay of the transverse magnetisation can be represented as the sum of up to *N* exponential decays as shown in (5).

5 $$ \kern2em S\left({t}_j\right)-e\left({t}_j\right)=\sum \limits_{i=1}^N{A}_i\ {\mathrm{e}}^{-\frac{t_j}{T_{2i}}},\kern2em N=1,\kern0.5em 2 $$

Whittall and MacKay in [22] stated that mono or bi-exponential analysis of materials with different T2 time constants is relatively accurate as opposed to more complex systems with *N* ≥ 3, where the T2 relaxometry becomes a nontrivial problem. Thus, our analysis was based on searching a maximum of two components. Every voxel curve was fitted twice in Eq. (5) for all *N* (*N* = 1, 2) meaning both mono exponential and biexponential fit as a preparatory step by using non-linear least squares. The number of exponentials was determined by the highest ${\overline{R}}^2$ described in the “Goodness of fit” section.

Non-linear least squares have the advantage of passing arguments such as the bound constraints for each variable for optimisation. In our case, the optimisation of (5) was succeeded with *A*_*i*_  ( *i* = 1,  2) in the range of 0 to 2000 and *T*_21_ ∈ [15, 120] ms and *T*_22_ ∈ [120.1 2500 ] ms. Additionally, in the case of *N* = 1, the *T*_21_ range was set to [15,7000] ms to account for slowly relaxing free water compartments. The choice of *A*_*i*_ was initially free (Levenberg Marquardt Algorithm [23] without bound constraints), and then the results obtained by this method were used to determine the bound’s range for more efficient fitting. The trust region reflective algorithm of the scipy.optimize.least_squares (www.scipy.org) was used in order to extract *A*_*i*_ and *T*_2*i*_ values from the raw relaxometry data. The intervals for T_2*i*_ were determined firstly by sequence limitations to detect very short T2 solid or tightly bound components and secondly by reported T2 values in the literature [24–27] for weakly bound and free water components. To avoid local minima, the fitting process for every voxel and model was performed 20 times having as initial starting points equally distributed within the range of each parameter bound.

### Goodness of fit

Having an analytical form of the model fitted to the data, the adjusted *R* squared (${\overline{R}}^2$) can be computed in order to acquire information about the goodness of fit. ${\overline{R}}^2$ is a generalised metric based on the *R* squared (*R*^2^), and its value is always less than or equal to that of *R*^2^ ∈ [0, 1]. This metric was proposed to overcome the limitation of *R*^2^ concerning that its value increases when more explanatory variables are added to the model [28]. Therefore, ${\overline{R}}^2$ was considered more suitable for this study than *R*^2^ since it takes into account the number of data points (*K*) and the number of the explanatory variables (m *i.e*., *m* = 2 for *N* = 1 and *m* = 4 for *N* = 2) of the model function. Moreover, from Eqs. (6) and (7), it is made obvious that the residuals between the model function (*G*_i_) and the data (*y*_i_) are also taken into account. Index *i* stands for the number of the measured voxel data points.

6 $$ {\overline{R}}^2=1-\left(1-{R}^2\right)\frac{K-1}{K-m-1} $$

7 $$ {R}^2=1-\frac{\sum_{i=1}^k{\left({G}_i-\overline{y}\right)}^2}{\sum_{i=1}^k{\left({y}_i-\overline{y}\ \right)}^2} $$

**Fig. 2:**
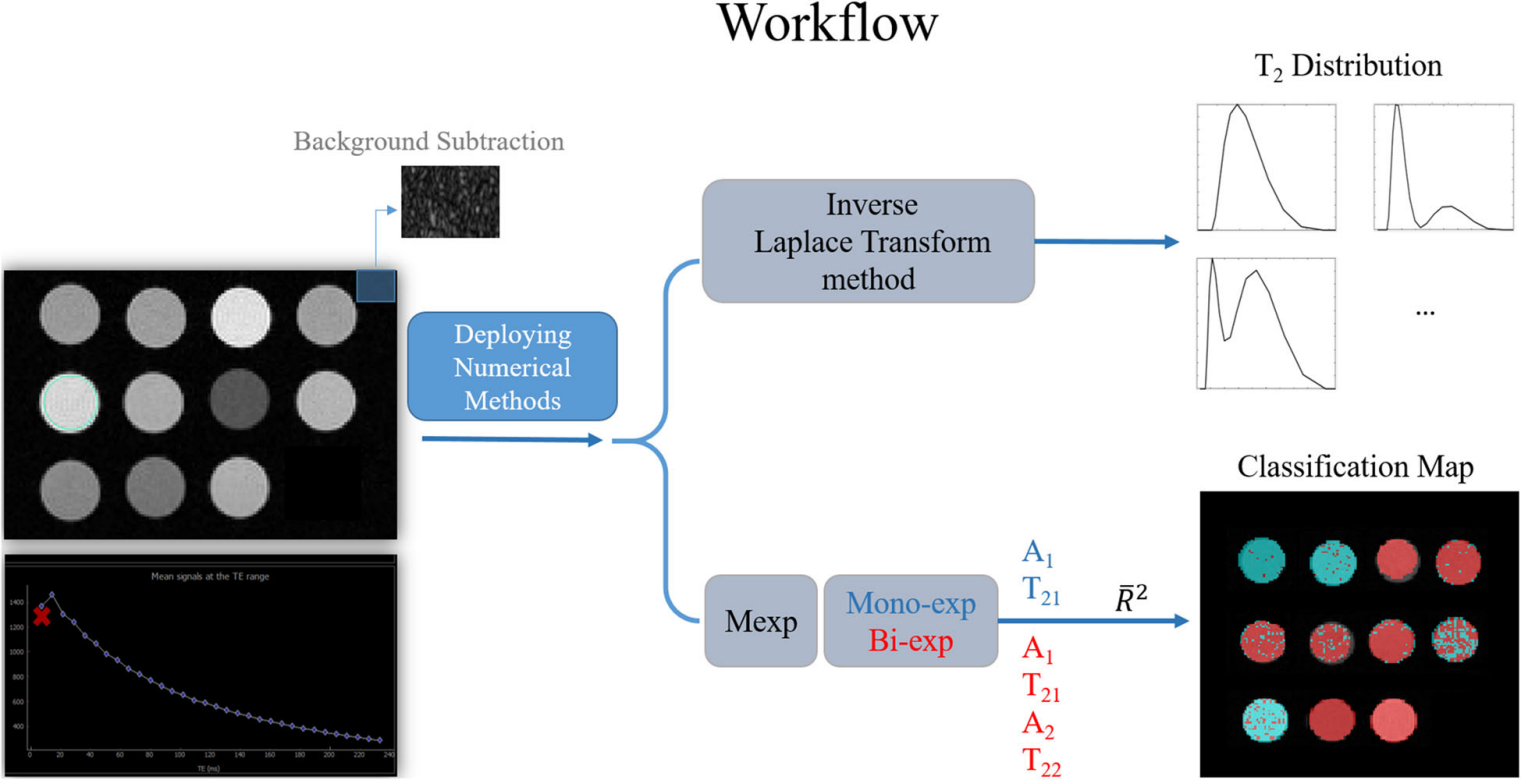
Study workflow with inverse Laplace transform and multiexponential methods

A graphical representation of the workflow used in this study is shown in Fig. 2.

### Quantitative T2 comparison by both methods

**Fig. 3:**
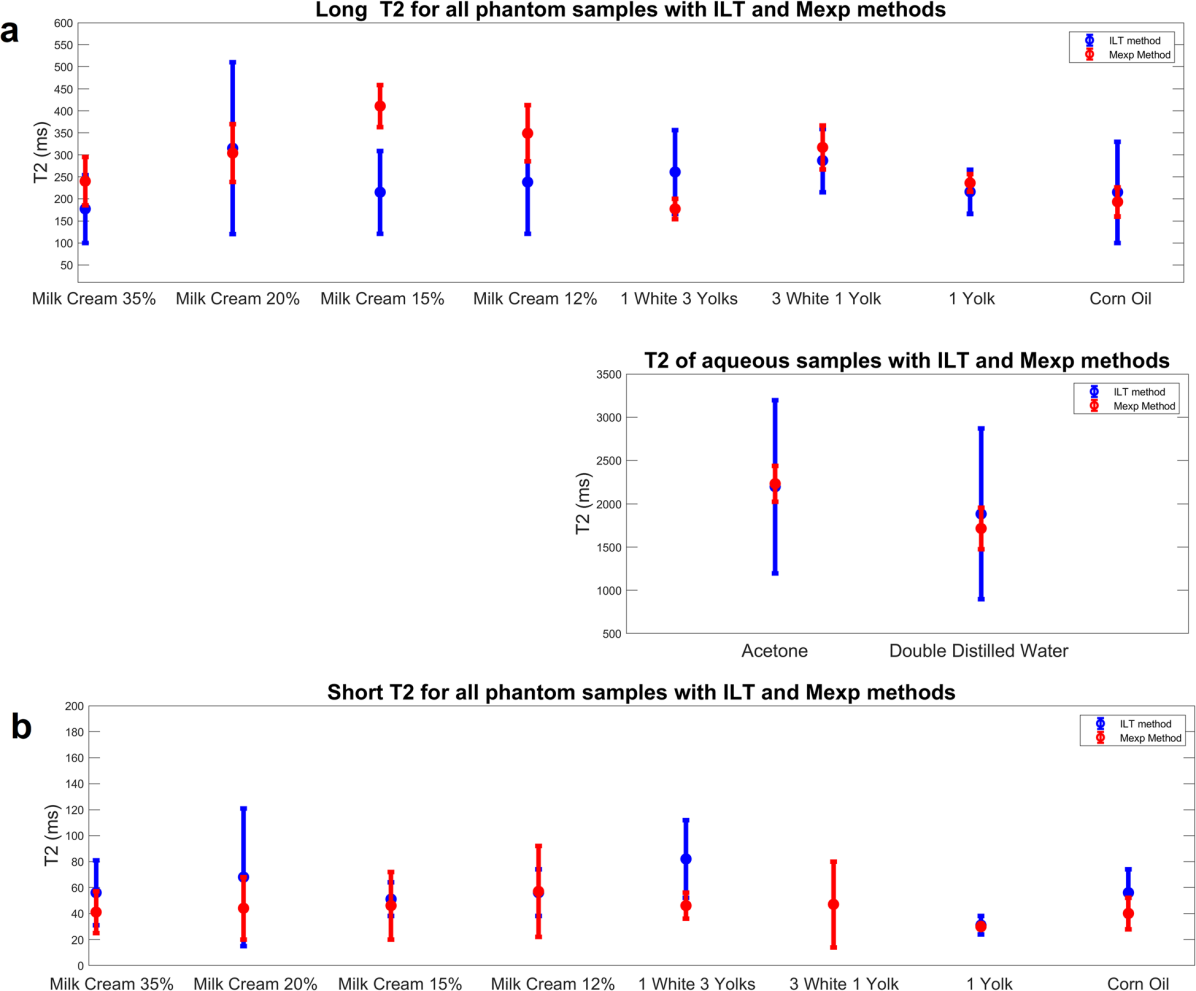
Graphical representation of long (**a**) and short (**b**) T2 components showing over all agreement between the two methods

Taking into account the different nature of the results obtained by each method (distribution vector for the ILT method as opposed to discrete *A*_i_ and *T*_2i_ values per voxel for the Mexp method), we proceeded to a further step in order to quantitatively compare the derived T2 values. More specifically, both short and long T2 distributions were separately fitted to a Gaussian distribution to obtain the mean and standard deviation for each mode. Furthermore, to quantify the relative contribution from the short T2 components, which is indicative of the fat content of the sample, the area under each Gaussian mode was calculated and is presented in Table 2. Accordingly, concerning the Mexp method, the evaluation of the fat content was based on the percentage of voxels characterised by biexponential T2 relaxation curve. Table 2 summarises the comparative results of T2 values as estimated by both methods as well as the contribution of the fatty component in the overall signal. Graphically, the distributions of T2 values (mean ± standard deviation) of both methods are presented as error bars in Fig. 3. Long *T*_2_ values from free water samples, double distilled water, and acetone were excluded from this figure depicting only samples with two components.

### Signal-to-noise ratio

An important magnetic resonance imaging parameter concerning quantification is image quality as assessed by signal-to-noise **(**SNR) values. For all sample positions, SNR was calculated based on the following equation at TE = 26.8 ms:

8 $$ SNR=\frac{mean(sample)}{std(Bg)} $$

where the numerator represents the mean value of all voxels in each vial, while the denominator stands for the standard deviation of the background signal.

## Results

**Fig. 4:**
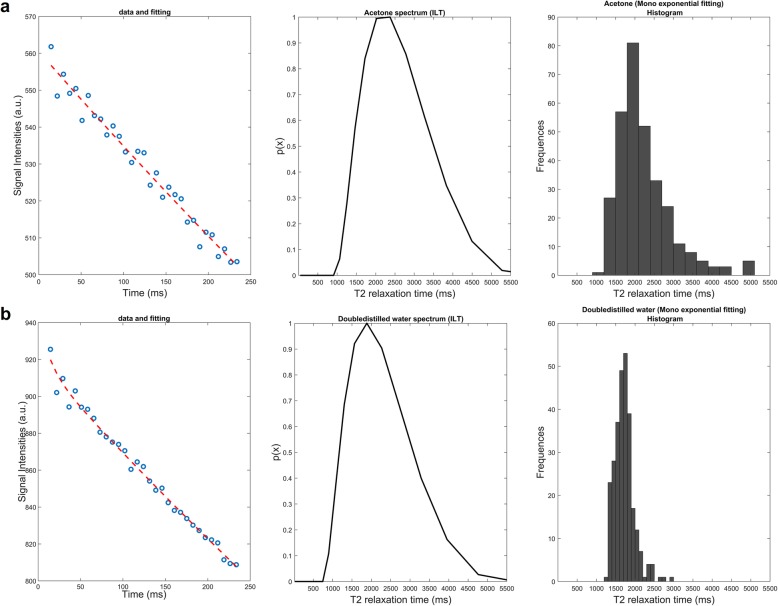
Apposition of both multiexponential (Mexp) and inverse Laplace transform (ILT) fitting on T2 decay curves of aqueous phantom samples. In each row the fitted data, ILT spectrum and T2 histogram with Mexp are presented for each sample. **a** Acetone. **b** Double distilled Water. Both methods exhibit pure monoexponential behavior

Aqueous samples, *i.e*., acetone and double distilled water, positioned in 1 and 2, respectively, showed ≥ 97% monoexponential behavior by the Mexp method (Fig. 4) and measured T2 values of the same order of magnitude (≈10^3^ ms) between the two methods.

**Fig. 5:**
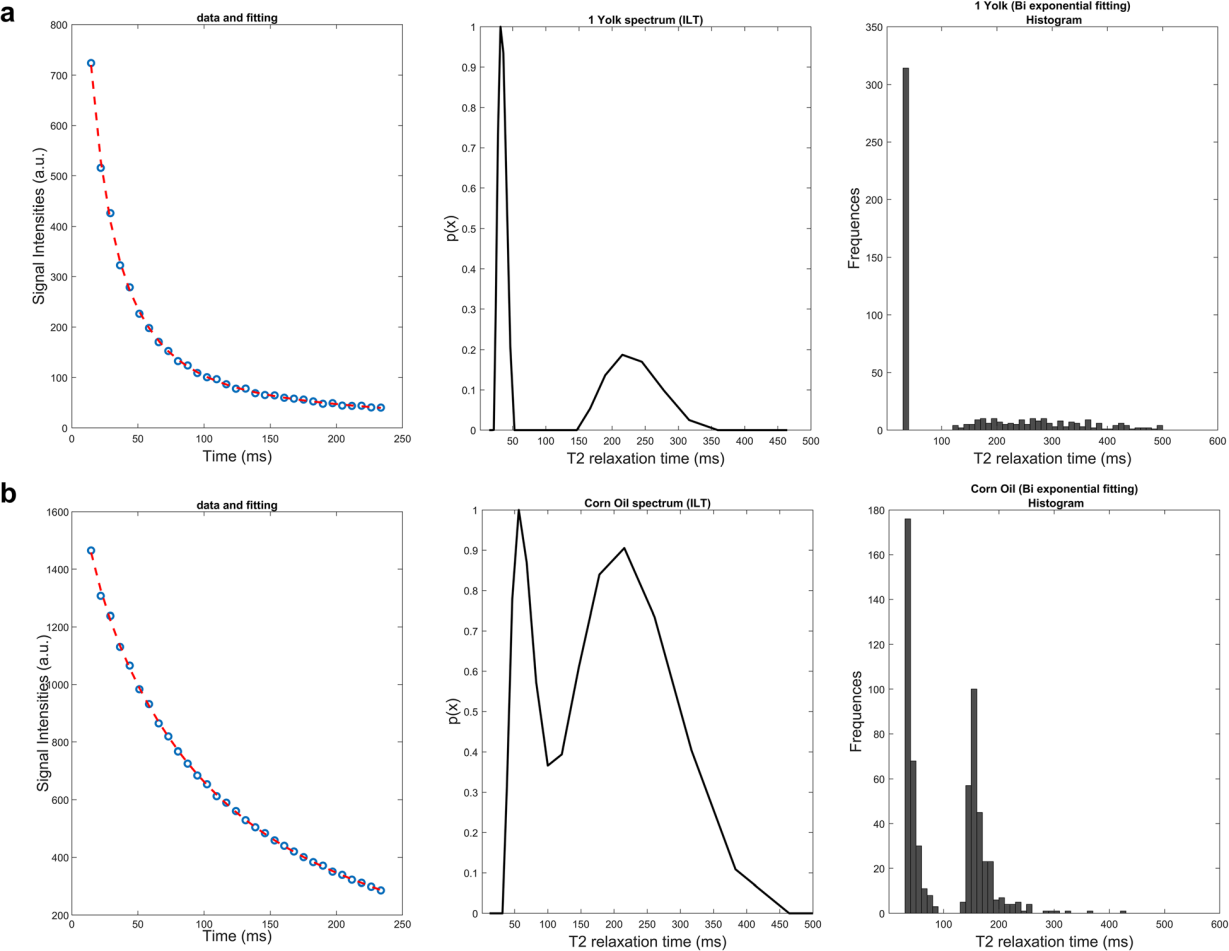
Apposition of both Mexp and ILT fitting on T2 decay curves of fat samples. In each row, the fitted data, inverse Laplace transform (ILT) spectrum and T2 histogram obtained with the multiexponential (Mexp) method are presented for each sample. **a** Egg yolk. **b** Corn oil. All voxels from both samples are characterised by biexponential behaviour

**Fig. 6:**
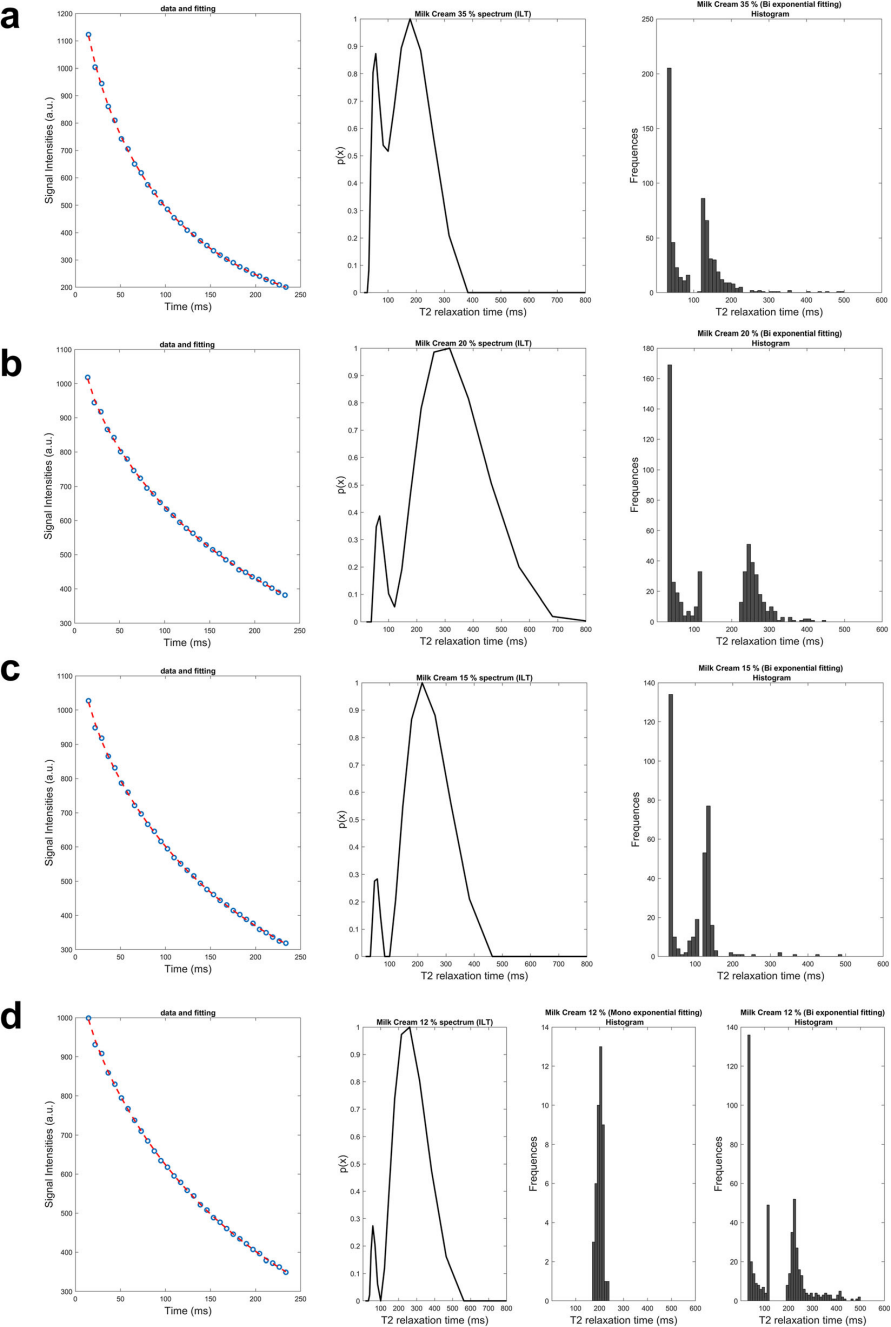
Apposition of both multiexponential (Mexp) and inverse Laplace transform (ILT) fitting on T2 decay curves of milk creams phantom samples. In each row, the fitted data, ILT spectrum and T2 histogram with Mexp are presented for each sample. **a** Milk cream 35% fat. **b** Milk cream 20% fat. **c** Milk cream 15% fat. **d** Milk cream 12% fat. The fourth sample (**d**) exhibits monoexponential contribution above 10% as calculated by Mexp, so both mono and bihistograms are shown

**Fig. 7:**
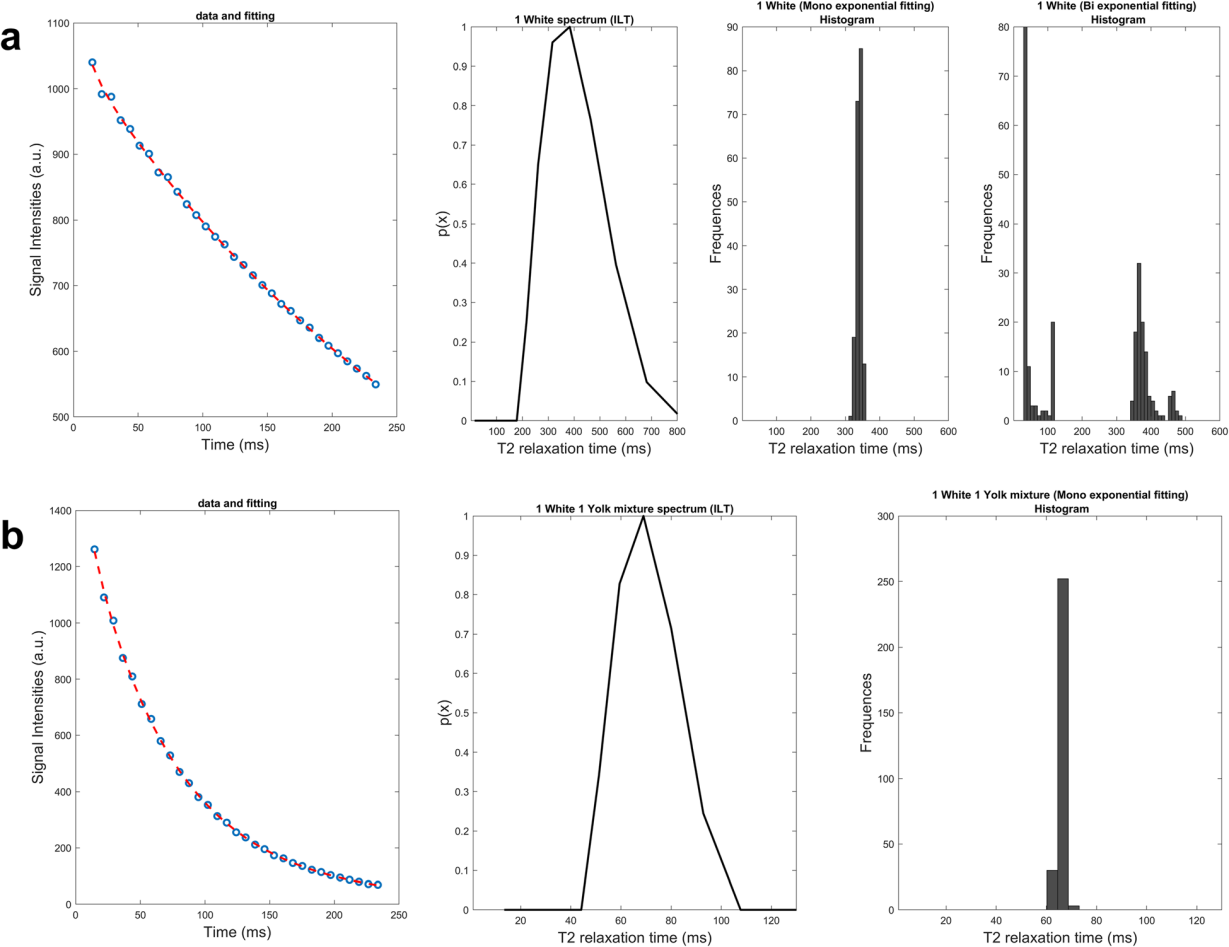
Apposition of both multiexponential (Mexp) and inverse Laplace transform (ILT) fitting on T2 decay curves of one egg white and a mixture of one egg white and one egg yolk. In each row, the fitted data, ILT spectrum, and T2 histogram with Mexp are presented for each sample. **a** One egg white. **b** one egg white and one yolk

**Fig. 8:**
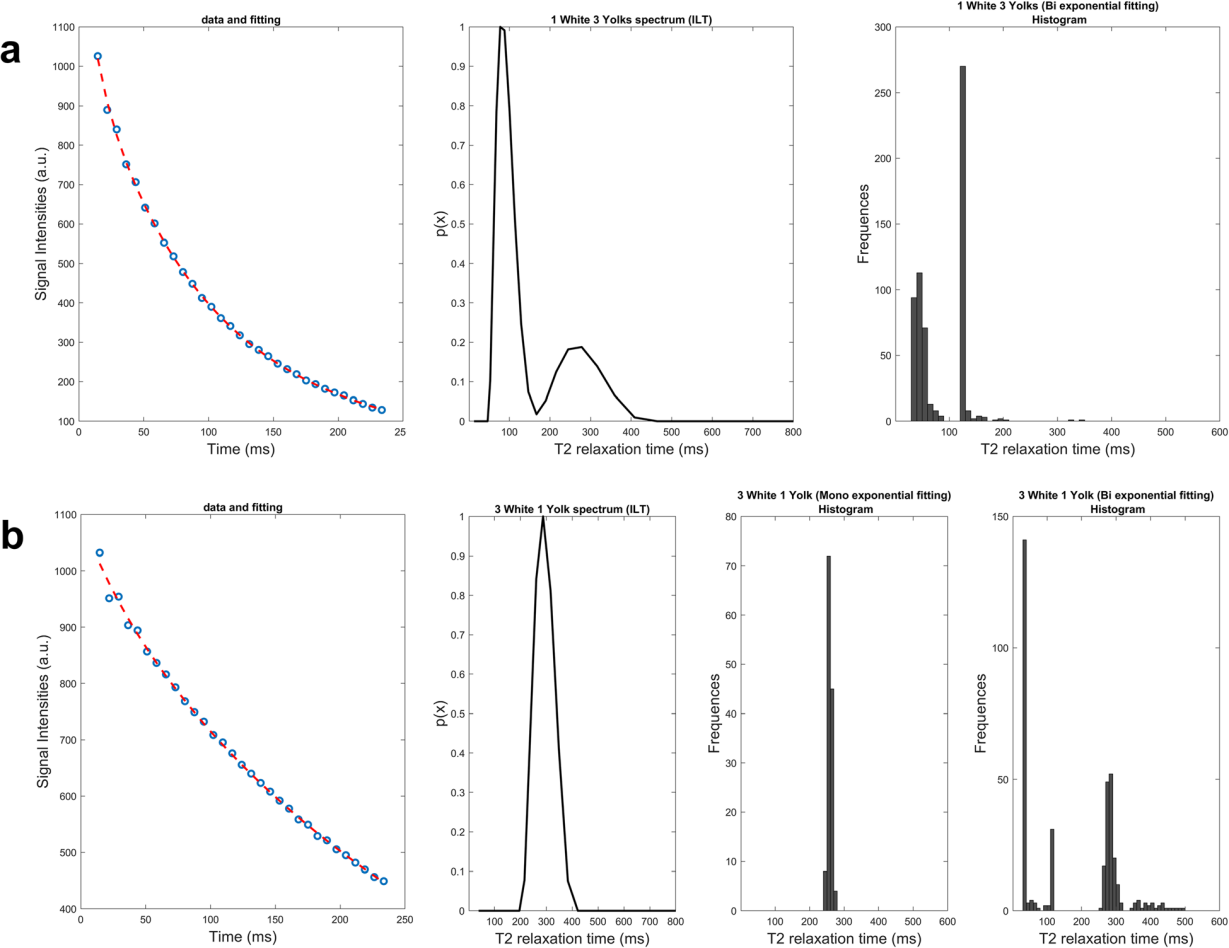
Apposition of both multiexponential (Mexp) and inverse Laplace transform (ILT) fitting on T2 decay curves of egg white and egg yolk mixtures. In each row, the fitted data, ILT spectrum, and T2 histogram with Mexp are presented for each sample. **a** One egg white. **b** Three egg whites and one yolk

The estimation of the number of components from samples of purely fatty composition (egg yolk and corn oil in positions 10 and 11, respectively) showed the presence of two different relaxation components for the 100% for the sample voxels from the Mexp method (Fig. 5). Mixtures of aqueous and fatty composition were presented in Figs. 6, 7, and 8. Specifically, milk creams with different fat fractions, namely, 35%, 20%, 15%, and 12% present in positions 3, 4, 5, and 6, respectively (Fig. 1), showed dominance of the biexponential model with variable degree of voxel percentage. In more detail, an increasing fraction of biexponential dominance was observed as fat fraction increased, namely from 100% biexponential dominance for milk cream 35% reduced to 85% biexponential dominance for the thinner milk cream of 12% (Table 1). Accordingly, the area of the shorter T2 component distribution as extracted from ILT varies with fat content, as it seems to progressively decrease with decreasing fat content (3.3% for milk cream with 12% fat, rising to 17.2% short T2 contribution for milk cream with 35% fat). For all milk creams, the ILT method showed bimodal distributions (Fig. 6). A not negligible (> 10%) monoexponential contribution from the thinnest milk cream of the phantom was found as calculated by the Mexp method.

**Table 1:** Multiexponenatial T2 analysis

Phantom study					
Phantom samples	Mono/biexponential prevalence (%)	*A*_1_ ± SD (a.u.)	T2_1_ ± SD (ms)	*A*_2_ ± SD (a.u.)	T2_2_ ± SD (ms)
Acetone	98/2	532 ± 20	2,231 ± 206	–	–
Double distilled water	97/3	893 ± 24	1,716 ± 240		
Milk cream 35%	0/100	519 ± 218	41 ± 16	794 ± 236	240 ± 55
Milk cream 20%	5/95	339 ± 246	44 ± 24	871 ± 261	304 ± 66
Milk cream 15%	10/90	409 ± 352	46 ± 26	830 ± 374	411 ± 48
Milk cream 12%	15/85	1,012 ± 47	200 ± 13	–	–
		313 ± 208	57 ± 35	764 ± 225	349 ± 64
One white, three yolks	10/90	1,115 ± 43	91 ± 2	–	–
		518 ± 141	46 ± 10	674 ± 152	177 ± 23
Three whites, one yolk	40/60	1,021 ± 48	258 ± 5	–	–
		169 ± 115	47 ± 33	910 ± 154	317 ± 50
One white, one yolk	91/9	1,511 ± 71	65 ± 2	–	–
One yolk	0/100	980 ± 36	30 ± 2	43 ± 15	236 ± 20
Corn oil	0/100	666 ± 183	40 ± 12	1,044 ± 10	193 ± 33
One white	60/40	1,034 ± 31	339 ± 7	–	–
		123 ± 74	50 ± 33	954 ± 86	395 ± 62

*a.u.* Arbitrary units, *A*_*1*_ Relative amplitude of the short T2 component, *A*_*2*_ Relative amplitude of the long T2 component, *T2*_*1*_ Short relaxation time, *T2*_*2*_ Long relaxation time, *SD* Standard deviation

Mixtures of egg whites and yolks (Figs. 7 and 8) also exhibited biexponential voxel dominance. In more detail, the sample containing a mixture of one egg white and three yolks had monoexponential/biexponential voxel dominance ratio of 10/90% while at the other end a mixture of increased aqueous component (three egg whites, one yolk) had a ratio of 40/60%. The mixture of one white and one yolk had 91% monoexponential dominance. For the mixture of three egg whites and one yolk, the ILT method failed to recognise the fat contribution and showed a monoexponential distribution while the Mexp method shows 60% biexponential behaviour.

Driven by this inconsistency of the two methods, we proceeded to the analysis of another sample of low fat composition (egg white) where again the Mexp method found significant biexponential contribution which the ILT could not identify showing a monoexponential distribution. All the aforementioned comparative results are presented in Table 2. A metric for image quality is presented in Table 3 with SNR values for all phantom samples.

**Table 2:** Comparative representation of short and long T2 values and fat content as derived from the short T2 contribution (ILT method) or biexponential contribution (Mexp method)

Samples	Phantom position	Mean T2 (inverse Laplace transform)			Mean T2 (multiexponential method)		
		Short T2 (ms) ± SD	Long T2 (ms) ± SD	Short T2 contribution	Short T_2_ (ms) ± SD	Long T2 (ms) ± SD	Biexponential contribution
Acetone	1	–	2,196 ± 1,066	0.0%	–	2,231 ± 206	2%
Double distilled water	2	–	1,884 ± 987	0.0%	–	1,716 ± 240	3%
Milk cream 35%	3	56 ± 25	177 ± 77	17.2%	41 ± 16	240 ± 55	100%
Milk cream 20%	4	68 ± 53	315 ± 195	5.2%	44 ± 24	304 ± 66	95%
Milk cream 15%	5	51 ± 13	215 ± 94	3.6%	46 ± 26	411 ± 48	90%
Milk cream 12%	6	56 ± 18	238 ± 117	3.3%	57 ± 35	349 ± 64	85%
One white, three yolks	7	82 ± 30	261 ± 95	44%	46 ± 10	177 ± 23	90%
Three whites, one yolk	8	–	287 ± 72	–	47 ± 33	317 ± 50	60%
One white, three yolks	9	69 ± 25	–		65 ± 2	–	9%
One yolk	10	31 ± 7	216 ± 50	30%	30 ± 2	236 ± 20	100%
Corn oil	11	56 ± 18	215 ± 115	20%	40 ± 12	193 ± 33	100%
One white	–	–	380 ± 200	–	50 ± 33	395 ± 62	40%

Short T2 contribution is the ratio of (area of short T2 distribution)/(area of short T2 distribution + area of long T2 distribution) and biexponential T2 contribution is the ratio of number of voxels classified as biexponential/number of monoexponential voxels + number of biexponential voxels

**Table 3:** Signal-to-noise values per sample

Phantom position	Signal-to-noise ratio
1	65.83
2	71.73
3	90.31
4	81.96
5	85.04
6	82.66
7	82.54
8	83.69
9	108.16
10	61.13
11	118.01

## Discussion

Transverse relaxation (T2) rate (spin dephasing) is a measure of the mobility of water molecules, which in turn is indicative of confounding structures or the presence of other macromolecules that bind to the water dipole molecule or of less mobile protons from larger molecules, such as lipids [7].

We obtained phantom results using a Mexp method with no *a priori* assumption on the number of distinct T2 compartments, and then were looked in comparison to the well-established ILT method for obtaining T2 spectrum. Considering that the aim of the study was to identify composition based on known patterns of relaxation for water or lipid compartments, we used samples of known composition with background knowledge on their relaxation properties.

The rationale for using ILT method for reference is its ability to detect the total number of components from a given dataset since the algorithm searches for all possible distributions in a given range. On the contrary, trying to solve an ill-posed problem, the Mexp method is constrained to a maximum number of *N* = 2 exponentials. By comparing the results between the two methods, we may conclude that the number of exponentials (*N* = 2) was adequate since no result from the ILT method exhibit a third distribution. However, in the frame of a different acquisition protocol, we cannot exclude the presence of a third exponential from the same samples.

In samples where ILT failed to identify the contribution of lipid compartments (mixture of three egg whites and one egg yolk), the fraction of biexponentially dominated voxels where low, as measured by Mexp method. Considering that we had a limited number of such voxels in our initial experimental set up, we also performed additional imaging at other low fat samples (egg white, Fig. 7) for purposes of confirmation of our hypothesis, stating that ILT failed to identify a distribution at the low T2 area when fat content was low. Mexp for the samples where ILT seemed to fail to identify a second curve in the spectrum measured a biexponential contribution of 60% for three whites and one yolk and 40% for the egg white sample.

Bounds necessary for fitting the biexponential function on our data where set based on results corresponding to body free moving fluids, such as cerebrospinal fluid (T2 > 2 s), and T2 values of water restricted within cellular structures, such as water particles between the lipid layers of the myelin sheath or intermyobrillar or myofibrillar protons (15 ms < T2 < 80 ms) or protons from fatty acid chains, while other components with more rapid relaxation, such as protons residing at the vicinity of the macromolecules (T2 < 0.03 s) could not be identified under this clinical sequence setting [24–26, 29, 30]. Based on these reported results, we set the ranges of slow and fast relaxing component.

Both aqueous samples analysed by both methods exhibited the expected monoexponential distribution (ILT) or single exponential decay (Mexp) and also exhibited T2 values of the order of 10^3^ ms, implying unrestricted proton motion (Fig. 1).

Corn oil was used as it closely matches the spectrum and longitudinal relaxation times of subcutaneous abdominal fat [30] and was best described by bimodal T2 distributions and biexponential model for 100% of the voxels [12]. Dairy cream of variable fat content was used as they provide a mixed aqueous and fatty environment with measurable contribution from each component with the short T2 component being indicative of the fat fraction [31]. Egg white exhibits two-component decay with intermediate and long T2 times. Meanwhile, yolk is generally characterised with triexponential decays, with short, intermediate, and very long decay times. Experimental results have shown that the intermediate component of yolk could be attributed to lipids [32]. For all samples with mixed composition, it can be observed that the percentage of biexponential voxels by Mexp is increased as the fatty component is more abundant.

Another important observation to discuss is the wider dispersion of the ILT-derived distribution vector which is evident by the length of error bars (Fig. 3). This was attributed to the combined effect of the broad search area for T2 values since there is lack of *a priori* knowledge of the expected values (from 10 to 10^4^), and the limited number of available measured data points (33 in our protocol) resulting in large search steps. This in turn reduces the precision of the T2 distribution shape. In the hypothesis of additional points added by the user to circumvent T2 broadening, this could confound the ability to solve the problem since it is both ill-posed and under-determined. It is important to note that the different assumptions and methods of ILT and Mexp do not allow for a comparison between the derived results. However, this will not limit the validity in case of a clinical Mexp T2 relaxometry application since the results will be obtained in a well-defined single methodology and not comparatively to any other method.

One of the major challenges related to T2 relaxometry is the acquisition protocols with adequate SNR to ensure accurate and repeatable measurements. Compromised SNR results in peak broadening and confounds accurate multi exponential fitting. In an experimental simulation of a three pool model, an SNR of the order of 500 was reported for accurate detection (> 80% of samples) of three exponential curves, while an SNR of 150 detected only 30% [33]. However, such SNR levels are very challenging in a clinical setting, in terms of spatially localised signal and acquisition timing. In our study, SNR ranged from 60 to 120, depending on the sample. In the frame of a phantom study, we could have prolonged the acquisition time to achieve better SNR values. However, since this work serves as a preliminary study for the translation of our results to a patient study, we kept the acquisition time to a clinically acceptable level.

In conclusion, this work apposed results from a proposed Mexp method with no *a priori* assumptions on the number of components with results from the well-established ILT method. Agreement between the two methods suggests the possible use of Mexp to provide valuable tissue specific information stemming from a microscopic tissue scale as an adjunct to conventional radiological assessment of the complex tissue microenvironment with the added advantage of voxelwise mapping. T2 relaxometry has a wide range of applications in medical and other fields, and the proposed method can be useful when there is insufficient *a priori* knowledge for the relaxation pattern of the sample, as is for the characterisation of heterogeneous neoplasms, brain tissue integrity, cartilage degeneration, and also for the study of benign and malignant adipocytic tumours.

## Data Availability

All DICOM images from T2 relaxometry acquisition are available upon request to the corresponding author.

## Abbreviations

ILT: Inverse Laplace transform
Mexp: Multiexponential
$$ {\overline{R}}^2 $$: Adjusted *R*^2^
ROI: Region of interest
SNR: Signal-to-noise ratio
